# Automated Planning for Prostate Stereotactic Body Radiation Therapy on the 1.5 T MR-Linac

**DOI:** 10.1016/j.adro.2021.100865

**Published:** 2022-02-12

**Authors:** Stefania Naccarato, Michele Rigo, Roberto Pellegrini, Peter Voet, Hafid Akhiat, Davide Gurrera, Antonio De Simone, Gianluisa Sicignano, Rosario Mazzola, Vanessa Figlia, Francesco Ricchetti, Luca Nicosia, Niccolò Giaj-Levra, Francesco Cuccia, Nadejda Stavreva, Dobromir S. Pressyanov, Pavel Stavrev, Filippo Alongi, Ruggero Ruggieri

**Affiliations:** a Department of Advanced Radiation Oncology, IRCCS, Sacro Cuore Don Calabria, Verona, Italy; b Faculty of Physics, Sofia University “St. Kliment Ohridski,” Sofia, Bulgaria; c Elekta AB, Stockholm, Sweden; d University of Brescia, Brescia, Italy

## Abstract

**Purpose:**

Adaptive stereotactic body radiation therapy (SBRT) for prostate cancer (PC) by the 1.5 T MR-linac currently requires online planning by an expert user. A fully automated and user-independent solution to adaptive planning (mCycle) of PC-SBRT was compared with user's plans for the 1.5 T MR-linac.

**Methods and Materials:**

Fifty adapted plans on daily magnetic resonance imaging scans for 10 patients with PC treated by 35 Gy (prescription dose [D_p_]) in 5 fractions were reoptimized offline from scratch, both by an expert planner (manual) and by mCycle. Manual plans consisted of multicriterial optimization (MCO) of the fluence map plus manual tweaking in segmentation, whereas in mCycle plans, the objectives were sequentially optimized by MCO according to an a-priori assigned priority list. The main criteria for planning approval were a dose ≥95% of the D_p_ to at least 95% of the planning target volume (PTV), V_33.2_ (PTV) ≥ 95%, a dose less than the D_p_ to the hottest cubic centimeter (V_35_ ≤ 1 cm^3^) of rectum, bladder, penile bulb, and urethral planning risk volume (ie, urethra plus 3 mm isotropically), and V_32_ ≤ 5%, V_28_ ≤ 10%, and V_18_ ≤ 35% to the rectum. Such dose-volume metrics, plus some efficiency and deliverability metrics, were used for the comparison of mCycle versus manual plans.

**Results:**

mCycle plans improved target dose coverage, with V_33.2_ (PTV) passing on average (±1 SD) from 95.7% (±1.0%) for manual plans to 97.5% (±1.3%) for mCycle plans (*P* < .001), and rectal dose sparing, with significantly reduced V_32_, V_28_, and V_18_ (*P* ≤ .004). Although at an equivalent number of segments, mCycle plans consumed moderately more monitor units (+17%) and delivery time (+9%) (*P* < .001), whereas they were generally faster (–19%) in terms of optimization times (*P* < .019). No significant differences were found for the passing rates of locally normalized γ (3 mm, 3%) (*P* = .059) and γ (2 mm, 2%) (*P* = .432) deliverability metrics.

**Conclusions:**

In the offline setting, mCycle proved to be a trustable solution for automated planning of PC-SBRT on the 1.5 T MR-linac. mCycle integration in the online workflow will free the user from the challenging online-optimization task.

## Introduction

Automated planning for radiation therapy (RT), that is, plan generation by the treatment planning system (TPS) without any user intervention during optimization (autoplanning), is a longstanding aim of RT (eg, since 1998^1^), both to speed the planning process and to reduce interplanner variability.^2^ Current commercially available autoplanning solutions can be grouped into 3 main classes^3^: knowledge-based model libraries (eg, RapidPlan by Eclipse TPS, Varian Medical Systems, Palo Alto, California), template-based algorithms (eg, AutoPlanning by Pinnacle^3^ TPS, Philips Medical Systems, Fitchburg, Wisconisn), and multicriterial optimization (MCO). In the a priori approach to MCO (eg, Monaco TPS, Elekta AB, Stockholm, Sweden) a single pareto-optimal plan, as the clinically desired tradeoff among all treatment goals, is directly generated.^3^^,^^4^ As a further step toward automatization, a priori MCO can be combined with lexicographic optimization (eg, Erasmus-iCycle optimizer, Erasmus University, Rotterdam, Netherlands),^5^ where optimization criteria are distinguished between constraints, which cannot be violated, and objectives, with an assigned relative importance (or priority). During the iterative optimization, the objectives can be turned into constraints but without compromising the previously achieved constraints. The set of constraints and prioritized objectives for a specific treatment site and protocol defines a “wish list.” Applications of Erasmus-iCycle were reported for various anatomic sites such as head and neck,^6^^,^^7^ cervix,^8^ prostate,^9^ and lungs.^10^ More recently, Erasmus-iCycle was implemented into the Monaco TPS^11^, ^12^, ^13^ as “mCycle,” the main novelty being the adoption of the physical and radiobiological cost functions of Monaco into the lexicographic logic.

The recent introduction of linacs coupled with a magnetic resonance imaging (MRI) scanner has generated the need of fast and accurate planning for online adaptive RT (onART). On the 1.5 T MR-linac (Unity, Elekta AB), onART can be performed by 2 distinct workflows^14^: “adapt-to-position,” in which only translational shifts are corrected, and “adapt-to-shape” (ATS). In the ATS workflow, all interfraction setup errors such as translations, rotations, and organ deformations, as they appear on the predelivery MRI-scan and are translated into the daily recontoured target(s) and organs-at-risk (OARs), can be corrected by reoptimization starting from the fluence map. For prostate cancer (PC) stereotactic body radiation therapy (SBRT), typically given in 5 fractions, ATS workflow is generally the standard choice. The full involvement of an expert planner in such online optimization workflow, as previously detailed,^15^ is a complex task. Thus, aiming to the potential substitution of the human expert by a trustworthy autoplanning system, we performed this preliminary plan comparison study as the first verification, to our knowledge, of the feasibility of using mCycle for PC-SBRT on the 1.5 T MR-linac.

## Methods and Materials

### Patients and plans

Ten patients with low- to medium-risk localized PC treated on Unity by a 7 MV-FFF photon beam from October 2019 to January 2020, with a prescription dose (D_p_) of 35 Gy given in 5 fractions within 2 weeks, were selected for this institutional review board–approved retrospective dosimetry study (part of a prospective observational study, numbered 23748), which included informed consent from each patient and whose inclusion and exclusion criteria had been previously described.^16^

The intensity modulated radiation therapy (step-and-shoot) plans from the human planner (“manual”) conceived in this study were replanned from scratch by Monaco (5.59.13 research version, running on 2 Quadro-GV100 32GB Nvidia GPUs) on the 5 MRI scans and structure sets of the daily treatments, where the planning target volume (PTV) was obtained by isotropically expanding the clinical target volume (CTV) by 5 mm except by 3 mm posteriorly, by starting from the template of cost functions and parameter values of the original ATS plans. All plans computed here, similarly to our ATS plans detailed previously,^15^ were based on 16 angularly equispaced static fields for a total of less than 100 segments and optimized by fixing the electron density to 1.0 for each tissue, but for the bone tissues (femoral heads, iliac wings, and sacrum), with bulk densities obtained from computed tomography. The segmentation settings were 8 mm as a minimum segment width, 5 cm^2^ as a minimum segment area, “high” for fluence smoothing, and 9 as the minimum monitor units (MUs) per segment. The dose was computed by the GPUMCD Monte Carlo dose calculation engine, which takes into account the 1.5 T magnetic field, with a 3 mm grid spacing and a 1% uncertainty per plan. Based on the same MRI scans and structure sets used for the 50 (5 fractions per each of 10 patients) manual plans, 50 mCycle plans were reoptimized from scratch (Monaco 5.59.13 res. v.) by using the same segmentation settings of the manual plans and according to the dosimetric criteria adopted for treatment, as detailed previously^15^ and consistent with a previous report.^17^ In summary, at least 95% of the D_p_ to at least 95% of the PTV (V_33.2_ ≥ 95%), although less than 107% of the D_p_ to the hottest 2% of the PTV (V_37.5_ ≤ 2%), had to be assured to the PTV. At the same time, V_33.2_ ≥ 95% had to be assured to the overlaps of the PTV with the rectum, bladder, and urethral planning risk volume (PRV, by 3-mm isotropic expansion), whereas V_32_ ≥ 95% could be accepted for rectal and bladder overlaps at online planning only. Requirements for the OARs were V_18_ ≤ 35%, V_28_ ≤ 10%, and V_32_ ≤ 5% to the rectum, V_35_ ≤ 1 cm^3^ to the rectum, bladder, urethral PRV, and penile bulb, and D_1cc_ ≤ 20 Gy to the femoral heads, where D_1cc_ refers to the hottest 1 cm^3^.

In Figure E1 (supplementary materials), the translation made by the Monaco TPS of these dosimetric criteria for planning approval in terms of semaphoric scorecards (where green indicates “passed”), which is updated while the optimization is ongoing, is shown for an example plan.

### Multicriterial optimization

In the Monaco TPS, the optimization process consists of 2 phases: phase 1 is a fluence matrix optimization, whereas during phase 2 (segmentation), both the shape (by the pseudo-gradient descent algorithm) and weight of all the segments are optimized. Optimization of manual plans was performed in “constrained” mode, where specific OAR-related cost functions (constraints) necessarily meet their goals, whereas the cost functions for PTV dose coverage (objectives) will meet their goals only after the constraints have been satisfied. Multicriterial optimization acts by optimizing the weights of the OAR-related cost functions for the ones for which it was selected (second-order objectives) so as to decrease their relative isoeffect (ie, stressing their action) until the point at which they start to affect the PTV-related objectives. In our online (ATS) planning, for each OAR-related cost function, the MCO option was selected during phase 1, whereas it was deselected during phase 2. When in phase 2, if any cost function was still out of the threshold, we could force its convergence within the threshold by manual tweaking of the related weight.

### mCycle autoplanning

mCycle consists of a 2-pass automated lexicographic MCO fluence map optimization, where the objectives are sequentially optimized according to a user-assigned order of priority (wish list) and automatically constrained. During pass 1, any objective that is violating its assigned goal is then constrained to such same goal value, which leaves room for objectives with lower priority. The objectives that are instead overcoming their goals are then constrained to their achieved values. During pass 2, the objectives that satisfied their goal at the end of pass 1 are skipped, whereas the objectives that were still violating their goal at the end of pass 1 are optimized until to the lowest achievable (or an assigned “sufficient”) value is reached. Next, segmentation is then performed by Monaco with its MCO. The wish list we developed for PC-SBRT, after preliminary tweaking on 3 patients, is detailed in Table E1 in the supplementary materials.

### In-phantom dosimetric verification

To test the consistency between manual and mCycle in terms of accuracy of dose delivery, a comparison by γ-analysis^18^ between in-phantom measured and computed doses was conceived. A total of 20 plans, from 1 couple of manual/mCycle plans for each of the 10 patients, were recomputed in a phantom made by rectangular slabs of solid water (RW3, PTW GmbH, Freiburg, Germany) with an interposed array of ionization chambers (Octavius1500MR, PTW GmbH). For such recomputation, the 3-mm dose-grid step and the 1% statistical uncertainty per calculation of the original plans were maintained, whereas the gantry angle of any field was reset to zero. The γ-values were computed with Verisoft 7.2 (PTW GmbH) software by neglecting any pixel with a computed dose lower than 5% of the maximum dose for both thresholds: 3 mm (3%) and 2 mm (2%) in distance-to-agreement (mm) and in locally normalized relative dose-difference (%), respectively.

### Comparison metrics and statistics

Manual and mCycle plans were compared in terms of the same dose-volume metrics as used in the scorecards (Fig 1S in the supplementary materials). Target dose coverage was evaluated by the V_95%Dp_ (V_33.2_) and V_107%Dp_ (V_37.5_) to the PTV and to the PTV minus its overlaps with the rectum, bladder, and urethral PRV (PTV_OVLs). The V_33.2_ was used for the urethral PRV, too, because it was contoured within the PTV. The adopted metrics for the OARs were the absolute volume of V_35_ (cm^3^) to the rectum (r), bladder (b), urethral-PRV (u), and penile bulb (p), plus the fractional V_18_, V_28_, and V_32_ for the rectum only.

Manual and mCycle plans were also compared by metrics focused on delivery both in terms of efficiency, that is, the total number of MUs and segments, the optimization time, and the computed delivery (beam-on) time and in terms of accuracy (“deliverability”), that is, the passing rate (PR) of γ-index for test criteria of 3 mm (3%) and 2 mm (2%) with local dose normalization.

The comparison metrics were first tested for normality in each sample by a Lilliefors test. According to the results, each couple of samples was then compared for location of medians or means (the null hypothesis being 2 samples of equal median/mean values) by the nonparametric Wilcoxon rank sum test (*U* test) or by the parametric *t* test, respectively. Furthermore, a nonparametric Levene test was used to test the equality of variances (the null hypothesis being 2 samples of equal variances). Such hypothesis testing was performed in Matlab (v. R2015, The Mathworks Inc) at the 5% significance level to reject the null hypothesis.

## Results

Patient-specific interfraction average values of each conceived metric are reported in Table 1 for target-related metrics and in Table 2 for metrics related to the critical OARs (ie, rectum, bladder, and penile bulb); interpatient descriptive statistics are also included in Table 2. All values of such metrics from all the plans, with 2 slight exceptions for V_33.2_PTV by manual plans, satisfied our criteria for planning approval.

**Table 1:** Individual interfraction averages of dose metrics related to the targets as computed by mCycle versus manual plans*

	V_33.2_^PTV_OVLs^ %		V_37.5_^PTV_OVLs^ %		V_33.2_^u^ %		V_35_^u^ (cm^3^)		V_33.2_^PTV^ %		V_37.5_^PTV^ %	
Patient	mCycle	Manual	mCycle	Manual	mCycle	Manual	mCycle	Manual	mCycle	Manual	mCycle	Manual
1	97.6	96.8	0.8	0.7	100.0	100.0	0.09	0.07	96.6	95.8	0.7	0.7
2	96.8	96.1	0.3	0.8	100.0	100.0	0.06	0.06	95.9	95.5	0.3	0.8
3	97.3	96.1	0.3	0.5	99.9	100.0	0.12	0.09	97.4	95.3	0.3	0.5
4	98.0	96.9	0.4	1.5	100.0	100.0	0.07	0.27	95.8	94.8	0.4	1.4
5	98.8	96.2	0.9	0.9	100.0	99.5	0.14	0.04	98.9	95.8	0.9	0.8
6	99.2	96.0	0.5	0.4	100.0	100.0	0.10	0.02	98.8	95.7	0.6	0.3
7	97.8	96.0	0.5	0.9	100.0	100.0	0.05	0.09	97.9	95.3	0.5	0.9
8	97.2	96.7	0.3	0.6	100.0	100.0	0.09	0.08	97.5	96.0	0.3	0.5
9	97.2	96.3	0.4	0.2	100.0	100.0	0.18	0.04	96.4	94.9	0.4	0.2
10	99.4	98.2	0.6	0.2	100.0	100.0	0.15	0.06	99.5	98.3	0.6	0.2
Mean	***97.9***	***96.6***	***0.5***	***0.7***	***100.0***	***100.0***	***0.11***	***0.08***	***97.5***	***95.7***	***0.5***	***0.6***
SD	*0.9*	*0.7*	*0.2*	*0.4*	*0.0*	*0.1*	*0.04*	*0.07*	*1.3*	*1.0*	*0.2*	*0.4*
Minimum	*96.8*	*96.0*	*0.3*	*0.2*	*99.9*	*99.5*	*0.05*	*0.02*	*95.8*	*94.8*	*0.3*	*0.2*
Maximum	*99.4*	*98.2*	*0.9*	*1.5*	*100.0*	*100.0*	*0.2*	*0.3*	*99.5*	*98.3*	*0.9*	*1.4*
p_1_ value	***<.001****^†^*		*.671*		*.999*		*.081*		***<.001****^†^*		*.671*	
p_2_ value	***.006****^†^*		*.379*		*NA*		***.009****^†^*		*.481*		***<.001****^†^*	

*Abbreviations:* V*_x_* % = minimum percentage volume to get ≥*x* (Gy); V*_y_* (cm^3^) = maximum absolute volume to get ≥*y* (Gy); SD = standard deviation; *NA* = not applicable; *PTV* = planning target volume

⁎ Targets were the PTV minus overlaps (PTV_OVLs), the urethral (u) planning risk volume (PRV), and the PTV. Interpatient statistics and *P* values from hypothesis testing including all 50 plans are reported, with p_1_ and p_2_ referring to tests comparing the medians (*U* tests) and the SDs (Levene tests), respectively. Outlined values for V_33.2_% are lower than acceptance criteria (95%). Italicized values are used for interpatient statistics, with mean values in bold. Rejection of the null hypothesis at the 5% significance level is indicated with boldface and ^†^.

**Table 2:** Individual interfraction averages of dose metrics related to the organs at risk, as computed by mCycle versus manual plans*

	V_35_^r^ (cm^3^)		V_32_^r^ %		V_28_^r^ %		V_18_^r^ %		V_35_^b^ (cm^3^)		V_35_^p^ (cm^3^)	
Patient	mCycle	Manual	mCycle	Manual	mCycle	Manual	mCycle	Manual	mCycle	Manual	mCycle	Manual
1	0.00	0.00	1.4	2.3	4.8	6.9	14.2	18.6	0.01	0.11	0.00	0.00
2	0.00	0.00	1.4	3.0	4.6	7.5	14.3	19.0	0.00	0.00	0.00	0.00
3	0.00	0.04	1.5	2.6	4.5	6.4	13.1	16.9	0.00	0.00	0.00	0.00
4	0.01	0.00	3.2	4.0	8.0	9.0	19.5	20.3	0.01	0.02	0.00	0.00
5	0.01	0.00	1.1	1.0	3.4	3.8	12.6	15.9	0.04	0.00	0.00	0.00
6	0.07	0.06	0.9	0.9	2.8	3.0	10.4	13.0	0.01	0.00	0.00	0.00
7	0.00	0.00	1.0	1.5	3.8	5.5	13.4	18.4	0.02	0.00	0.00	0.00
8	0.00	0.00	1.0	1.7	4.0	5.2	15.2	16.5	0.00	0.00	0.00	0.00
9	0.00	0.00	1.2	2.3	4.2	6.5	13.5	17.8	0.02	0.00	0.00	0.00
10	0.01	0.02	0.1	0.2	0.8	1.0	8.4	10.4	0.01	0.00	0.00	0.00
Mean	***0.01***	***0.01***	***1.3***	***1.9***	***4.1***	***5.5***	***13.5***	***16.7***	***0.01***	***0.01***	***0.00***	***0.00***
SD	*0.02*	*0.02*	*0.8*	*1.1*	*1.8*	*2.3*	*2.9*	*3.0*	*0.01*	*0.03*	*0.00*	*0.00*
Maximum	*0.07*	*0.06*	*3.2*	*4.0*	*8.0*	*9.0*	*19.5*	*20.3*	*0.04*	*0.11*	*0.00*	*0.00*
p_1_ value	*.258*		***.004****^†^*		***.002****^†^*		***<.001****^†^*		***<.001****^†^*		*.999*	
p_2_ value	*NA*		***.006****^†^*		***<.001****^†^*		***<.001****^†^*		*.287*		*NA*	

*Abbreviations:* b = bladder; p = penile bulb; r = rectum; SD = standard deviation; *V_x_* % = minimum % volume to get ≥*x* (Gy); *V_y_* (cm^3^) = maximum absolute volume to get ≥*y* (Gy); *NA* = not applicable; *PTV* = planning target volume

⁎ Interpatient statistics and *P* values from hypothesis testing including all 50 plans are reported, with p_1_ and p_2_ referring to tests comparing the medians (*U* test) and the SDs (Levene test), respectively. Italicized values are used for interpatient statistics, with mean values in bold. Rejection of the null hypothesis at the 5% significance level is indicated with boldface and ^†^.

**Fig. 1:**
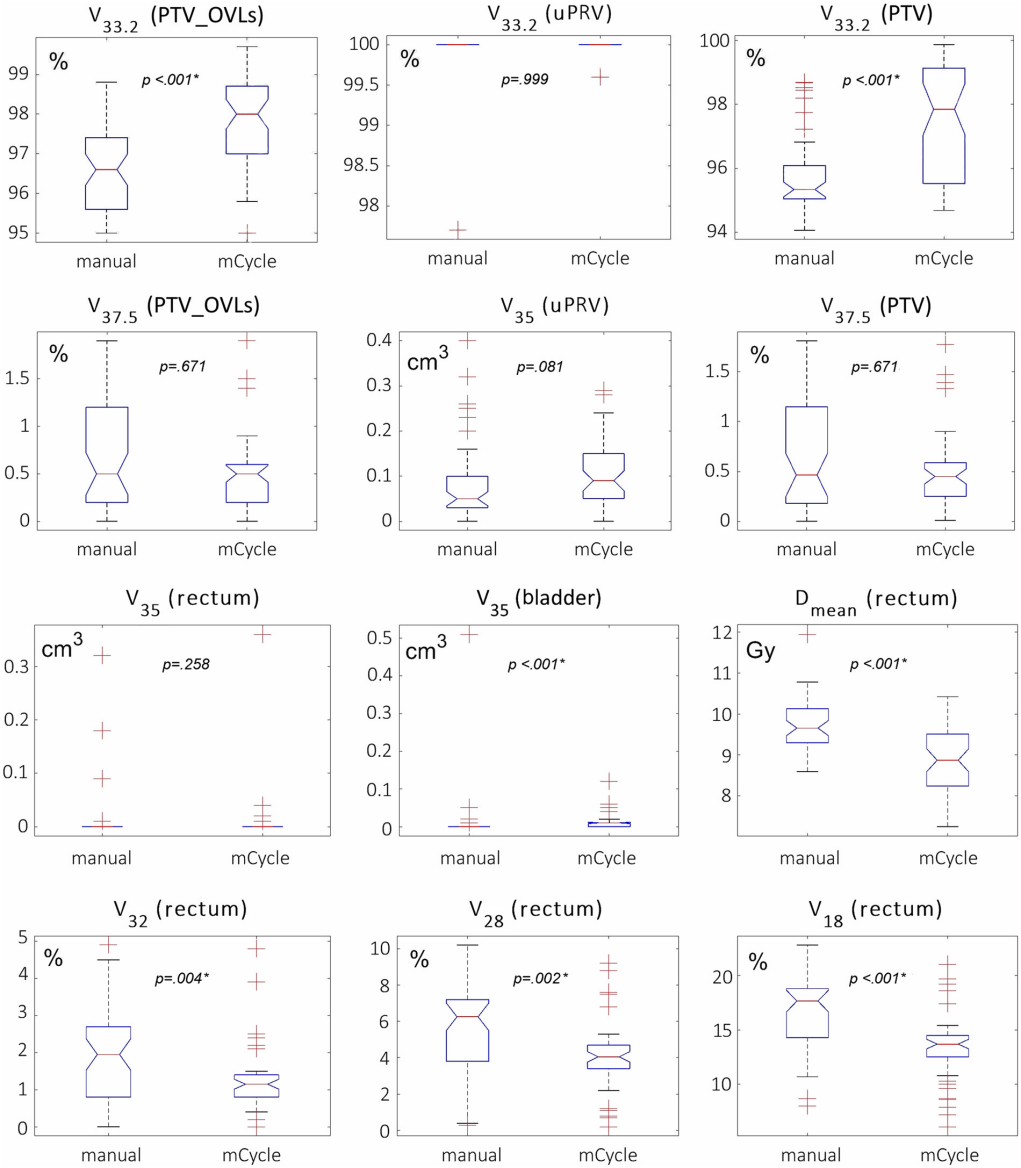
Box-and-whisker plots of the metrics computed from the 50 manual plans (left) and mCycle plans (right). In the first 2 rows are target (PTV_OVLs, uPRV, and PTV) dose coverage metrics (V_33.2_ %, V_37.5_ %, and V_35_ cm^3^). In the last 2 rows are the V_35_ (cm^3^) to the rectum and bladder, rectal mean dose, and fractional volume (V_32_ %, V_28_ %, and V_18_ %) metrics, where V_X Gy_ % (cm^3^) is the fractional (absolute) volume receiving a dose not less than X (Gy). The boxesare delimited by the 25th and 75th percentiles and medially crossed by the median value; the whiskers point to the 50th ± 1.57 (75th–25th)/√N percentiles, and the outliers are indicated by crosses. *P* values from *U* tests (*t* tests for the rectal mean dose only) hypothesis testing are overlaid (* indicates significance at the 5% level). *Abbreviations:* OVL = overlap with the planning risk volume; PTV = isotropic expansion of the CTV by 5mm except by 3mm posteriorly; uPRV = isotropic expansion of the urethra by 3mm; PTV_OVLs = PTV minus its overlaps with rectum, bladder, and uPRV.

The full distribution of values for such metrics over the whole sample of manual (left) and mCycle (right) plans is depicted in Figure 1 by box-and-whisker plots. In the first 2 rows are target-related (PTV_OVLs, uPRV, and PTV) metrics (V_33.2_%, V_35_ [cm^3^], V_37.5_%), whereas in the last 2 rows are the V_35_ (cm^3^) to both the rectum and bladder and the mean dose and fractional volume (V_32_%, V_28_%, V_18_%) metrics to the rectum. The same *P* values listed in Tables 1 and 2 (*p_1_*) from the *U* test, plus the *t* test for the rectal mean dose only, are overlaid in Figure 1 (the asterisk indicates significance at the 5% level). Statistically significant differences resulted for V_33.2_ (%) of both the PTV and PTV_OVLs for the mean dose and V_32_ (%), V_28_ (%), V_18_ (%) to the rectum, and V_35_ (cm^3^) to the bladder. Thus, mCycle plans improved not only target dose coverage, with V_33.2_ (PTV) passing on average (±1 SD) from 95.7% (±1.0%) for manual plans to 97.5% (±1.3%) for mCycle plans (*P* < .001), but also rectal sparing. In detail, V_32_^r^ passed on average from 1.9% (±1.1%) for manual plans to 1.3% (±0.8%) for mCycle plans (*P* = .004), V_28_^r^ passed from 5.5% (±2.3%) to 4.1% (±1.8%) (*P* = .002), and V_18_^r^ passed from 16.7% (±3.0%) to 13.5% (±2.9%) (*P* < .001). In Tables 1 and 2, the *P* values from the Levene test (*p_2_*) also are reported, similarly to Cilla et al,^19^ which are suggestive of a moderate reduction of interplan variability by mCycle plans in terms of both volumetric rectal sparing (V_32_, V_28_, V_18_) and control of the hotspots over the PTV (V_37.5_).

**Fig. 2:**
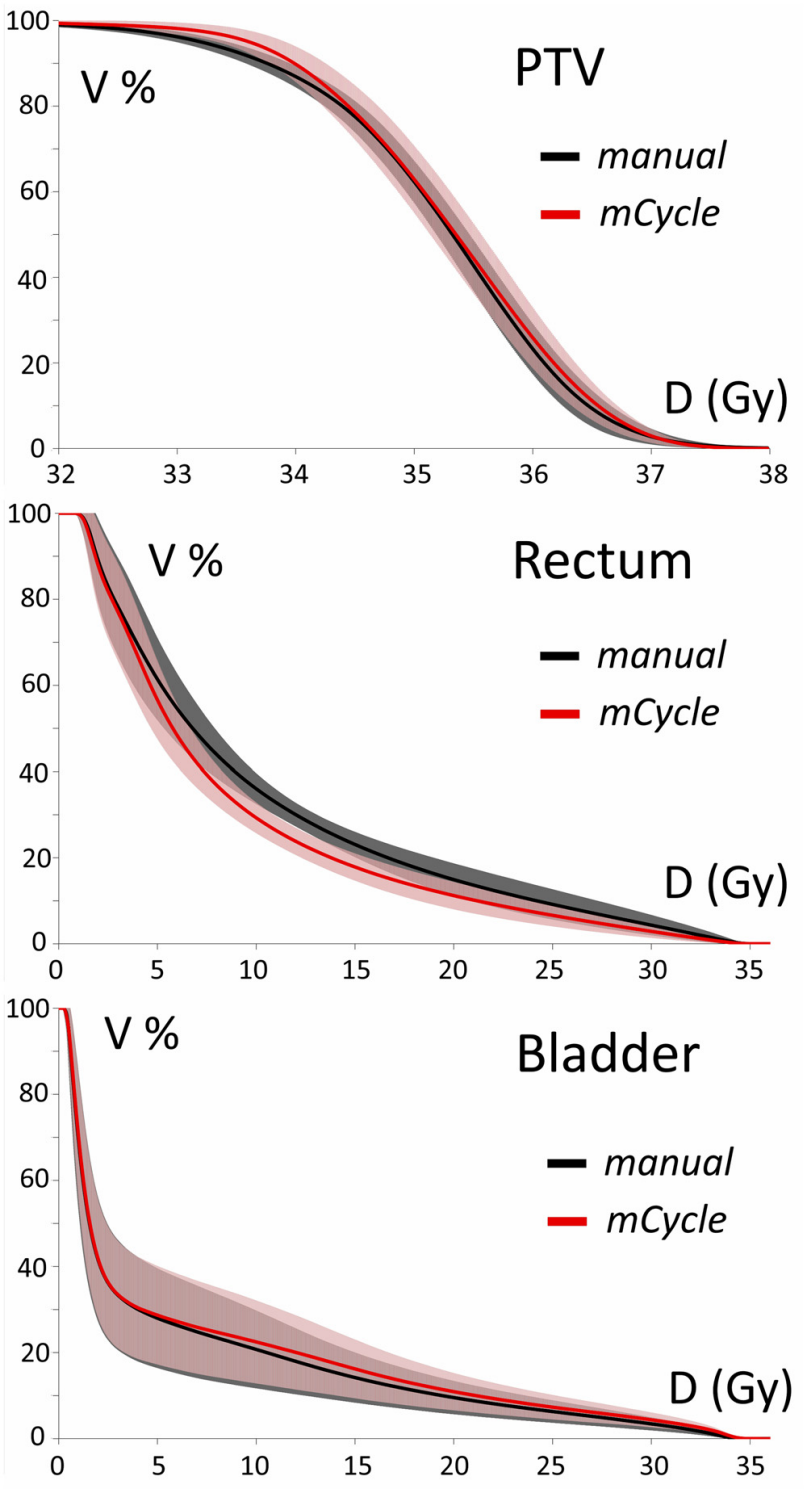
Average DVH curves (error bars indicate ±1 SD) for the PTV (first row), rectum (second row), and bladder (third row), by the 2 groups of 50 plans: mCycle (red) versus manual (black). *Abbreviations:* DVH, dose-volume histograms; PTV, planning target volume.

The average dose-volume histograms (DVH) curves (±1 SD error bars) for the PTV, rectum, and bladder by the 2 groups of 50 plans, mCycle versus manual, are depicted in Figure 2. Such curves make visible both the absence of any significant variation in mean dose to the bladder (third row) and the significant gains from mCycle plans in terms of target dose coverage at 95% of the D_p_ (first row) and of the mean rectal dose (second row), which was reduced to 8.9±0.7 Gy from the value of the manual plans (9.7±0.6 Gy) (*P* < .001; *t* test).

**Fig. 3:**
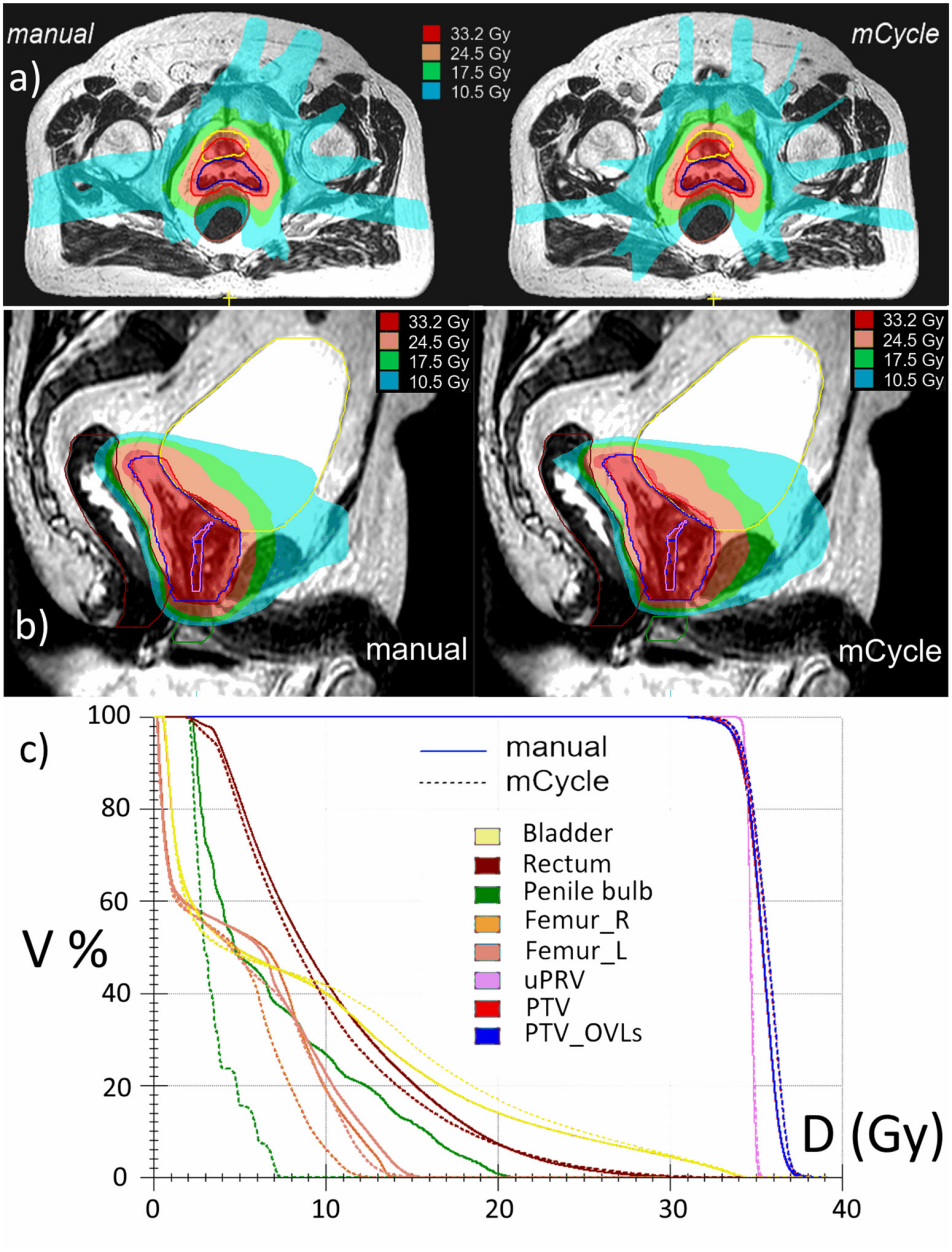
For a first representative patient, subplot *a* shows the improved isotropy in dose distribution at the intermediate dose levels, that is, 30% of the prescription dose (Dp) (cyan), 50% of the Dp (green), and 70% of the Dp (yellow), from mCycle plans (right) with respect to manual plans (left). For a second representative patient, subplot *b* shows the improved dose sparing of the penile bulb from the mCycle plan (right) with respect to the manual plan (left). The DVH curves for the second patient from mCycle and manual plans for the full set of constrained structures are shown in subplot *c. Abbreviation:* DVH, dose-volume histograms.

The dose distributions from the manual (left) and mCycle (right) plans of the same example patient are depicted in Figure 3 (subplot *a*) to show that manual plans may sometimes be associated with a slightly anisotropic dose distribution at the medium-to-low dose levels, that is, approximately 30% Dp (cyan). By defining a conformality index as the ratio of the volume (cm^3^) delimited by the 30% Dp (ie, 10.5 Gy) isodose line and the PTV volume (cm^3^), a significant difference resulted between manual (14.5±1.3) and mCycle (13.6±1.0) plans (*P* < .001; *U* test). As a likely consequence of such improved conformality, mCycle plans reduced the mean dose of the penile bulb from 6.8±3.3 Gy in the manual plans to 3.8±1.4 Gy (*P* < .001; *U* test), although at the clinically acceptable tradeoff of a not meaningful (*P* = .332; *U* test) and small increase of the mean dose to the bladder from 6.1±2.0 Gy to 6.2±2.2 Gy. This is shown for another example patient in Figure 3 (subplot *b*), where the full set of DVH curves from mCycle and manual plans is also reported (subplot *c*). These details, which are outside the set of necessary dose-volume constraints for planning approval, can be easily accounted for by an automated approach.

According to both efficiency and deliverability metrics, for the 2 groups of manual and mCycle plans, the observed mean values (±1 SD) and *P* values from hypothesis testing are reported in Table 3. Such results are referring to the whole sample of plans for MUs, segments, and delivery time and to a subgroup of 10 couples of plans in the case of the optimization time and the 2 deliverability metrics. These results suggest that mCycle optimization times, although with a large spread, were significantly shorter than the manual ones. Furthermore, the mCycle plans, although at an equivalent number of segments, were moderately more consuming of MUs (+17% on average, with + 25%, ie, 521 MUs, as a maximum) and delivery time (+9% on average, with +19%, ie, 1.5 min, as a maximum) consuming. However, despite such slightly increased complexity, no significant differences were found for both PR (3 mm [3%]) and PR (2 mm [2%]) deliverability metrics.

**Table 3:** Interpatient and interfraction averages and SDs of efficiency, as computed by mCycle versus manual plans

	MUs		Segments		Optimization time, s		Delivery time, s		PR (3 mm, 3%)		PR (2 mm, 2%)	
	mCycle	Manual	mCycle	Manual	mCycle	Manual	mCycle	Manual	mCycle	Manual	Mcycle	Manual
Mean	***2477***	***2118***	***81.2***	***80.4***	*470.6*	*676.6*	***505.8***	***463.3***	*99.5*	*99.8*	94.9	95.9
SD	*147.7*	*156.1*	*12.6*	*9.6*	*106.1*	*294.8*	*43.6*	*34.1*	*0.7*	*0.5*	3.4	2.9
p_1_ value	<.001*				<.019*				*.950*		*.432*	
P_2_ value			*.707*				<.001*					

*Abbreviations:* MU = monitor unit; PR = passing rate; SD = standard deviation.

These values include all 50 plans (bold values) in the case of MUs, segments, and delivery time and a subgroup of 10 couples of plans (20 plans) in the case of the optimization time and the 2 deliverability metrics. *P* values from hypothesis testing are reported0, where p_1_ is referring to a *U* test and p_2_ to a *t* test. Rejection of the null hypothesis at the 5% significance level is indicated with boldface and *.

## Discussion

Autoplanning is a current hot topic of development to reduce interplanner variability while generally improving plan quality.^3^ For PC-SBRT, the feasibility of autoplanning for even complex techniques as simultaneous integrated boost to the dominant intraprostatic lesion has been reported^20^, and as shown in Figure 2S (supplementary materials) as an example case, such feasibility also holds for the 1.5 T MR-linac by mCycle. For onART, such as magnetic resonance guided RT(MRgRT), the speed of the planning process, here including contouring, optimization, and plan evaluation, is crucial to reduce the risk of intrafraction motion, which might potentially compromise the benefit of daily adaptive RT.^15^ To this purpose, neural-network-based models have been proposed for automated segmentation^21^ or for an anatomy-based prediction of the daily dose distribution as a benchmark for a rapid evaluation of the adaptive plan quality.^22^ Furthermore, online adaptive replanning (eg, ATS on the 1.5 T MR-linac) is a complex procedure sensitive to the planner's expertise, which likely acts as a brake to the adoption of such a technique. Hence, there is interest in fast and accurate autoplanning software as an alternative to an expert planner. To this aim, we tested whether mCycle,^5^^,^^11^ whose tweaking requires time and expertise but has to be done once per class-solution,^6^, ^7^, ^8^, ^9^, ^10^ was a potential solution. This was done by focusing on PC-SBRT patients treated by daily ATS on the 1.5 T MR-linac and comparing mCycle plans with the manual ones by an expert human planner. A similar comparison for the 1.5 T MR-linac was done by Bijman et al^23^ for patients with rectal cancer treated by 50 Gy in 25 fractions, with some improved sparing of the OARs from autoplanning. The main difference between that study and the present study, other than Bijman and colleagues’ use of Erasmus-iCycle instead of mCycle, was the increased dose complexity that is required for pelvic treatments when passing from standard fractionation, where OAR sparing typically translates into mean dose reduction, to severe hypofractionation, where plan approval results from an optimal compromise between avoidance of the hotspot and dose coverage to the several overlaps of the target with the critical OARs (ie, rectum, bladder, urethra, and penile bulb). In another study on 1 patient with PC treated by 60 Gy in 20 fractions,^24^ an offline autoplanning solution for the 1.5 T MR-linac was tested, where an in-house made optimizer generated the fluence map, which was then passed as input to the standard segmenter of Monaco. The offline plan was then used as a reference plan in the daily ATS workflow; however, the usual optimization tools of online Monaco were adopted to generate any adaptive plan. The aim of the present study was instead to test in the offline setting if mCycle might become a valid autoplanning alternative to current human-supervised optimization tools of online Monaco.

The choice of an offline setting for mCycle testing was necessary because the online use of Monaco on Unity is restricted to certified clinical versions only. The optimization times we computed were then conditioned by the offline hardware resources (2 Quadro-GV100 32GB Nvidia GPUs). By now observing that the mean (±1 SD) ratio of the observed optimization times for mCycle over manual plans was 81% (±34%), we deduced that future online use of mCycle on Unity could reduce (by 19% on average) the current manual optimization times. This satisfied the first of our 2 requirements for reliable autoplanning for the 1.5 T MRlinac: the quickness.

According to our second expectation, that is, the accuracy of autoplanning, we compared the quality of mCycle versus manual plans in terms of both dose distributions and efficiency and deliverability metrics. In terms of dose distributions, plan quality from mCycle was never inferior to that of manual plans. mCycle (as shown in Tables 1 and 2) slightly improved the target dose coverage (V_33.2_) for both the PTV and PTV_OVLs while assuring improved dose sparing of the rectum at all dose levels (V_32_, V_28_, V_18_), as reflected by an approximately 8% reduction in the mean rectal dose. The control of the hotpsot (V_35_) to the rectum, bladder, and urethral PRV was instead equivalent for the mCycle and manual plans, although a negligible V_35_ to the penile bulb resulted for all plans, simply because these patients’ bulbs were not proximal to the high dose region. Furthermore, based on the analysis of interplan heterogeneity by the Levene test,^19^ slightly improved homogeneity from the mCycle plans resulted in terms of rectal sparing and target dose coverage, although manual plans, too, all being planned by the same person, were satisfactorily homogeneous. Such results are consistent with those of 2 previous studies on Erasmus-iCycle versus manual plans,^4^^,^^9^ both dealing with prostate volumetric modulated arc therapy by standard fractionation (eg, 78 Gy/39 fx), which reported equivalent target dose coverage metrics, whereas statistical significance was reached for rectal^4^^,^^9^ and bladder^4^ dose-sparing metrics, respectively.

In terms of efficiency and deliverability metrics, we found that an equivalent number of segments but slight increases in the total number of MUs (+17%, ie, 360 MUs on average and +25%, ie, 521 MUs maximum) and in the computed delivery time (+9%, ie, 0.8 minutes on average and +19%, ie, 1.5 minutes maximum), were associated with the mCycle plans. This is consistent with the reported correlation between improved rectal sparing and increased MUs when comparing mCycle versus manual plans.^4^ Increased MUs and computed delivery times were also reported when comparing mCycle with its precursor, Erasmus-iCycle.^13^ Such slight increases in computed MUs and delivery time from mCycle plans, as likely associated with an increased degree of modulation (but still reducible by tweaking of the segmentation parameters, if desired), was not associated with any significant degradation in deliverability metrics. All the in-phantom tested plans achieved a PR ≥ 90%, which is our minimum criterion for the clinical use of the plan.

With regard to the level of automation, 1 mCycle plan out of 50 required the user's intervention: the original wish list, which had been tweaked on 3 patients, had to be slightly retuned by also including such a fourth patient. We nonetheless believe that even the 49 of 50 successes based on our initial wish list represent quite a robust level of automation, because the anatomic variability included in our 50 MRI planning scans was wide. This resulted from our onART by ATS, which compensated for even greater intrapatient variability in bladder and rectal filling between sessions.

Overall, these results depict mCycle as a robust autoplanning system to support even nonexpert planners in the ATS workflow for PC-SBRT on the 1.5 T MR-linac, thus encouraging the adoption of fully adaptive MRgRT.

## Conclusions

In the offline setting, both manual and mCycle plans for PC-SBRT were clinically acceptable, with the mCycle plans never being inferior to the plans generated by a human expert. mCycle might hence be integrated into the online planning MRgRT workflow of the 1.5 T MR-linac, thus removing the current need for an expert planner during each treatment session.

## Appendix Supplementary materials

Image, application 1

Image, application 2

Image, application 3
