# Supplementary material for: Automated Planning for Prostate Stereotactic Body Radiation Therapy on the 1.5 T MR-Linac
Source: Adv Radiat Oncol. 2022 Feb 12;7(3):100865. doi: 10.1016/j.adro.2021.100865 (PMC8850203; doi:10.1016/j.adro.2021.100865)
Supplement: Supplementary file 1 [file mmc1.docx]

**Tab. 1**: mCycle wish-list for auto-planning of prostate SBRT at 35 Gy given in 5 fractions.

| Clinical (C) / Planning (P) Constraints | | | | | |
| --- | --- | --- | --- | --- | --- |
| *Type* | *Layering*  *Order* | *Structure* | *Cost Function*  *(parameter values)* | *Shrink margin (cm)* | *Goal* |
| C | 5 | External | Maximum Dose | 0.0 cm (*a,b,c,d*) | < 38 Gy |
| C | 6 | rectum | Parallel (32 Gy, *k* = 4) |  | < 4.5 % |
| C | 6 | rectum | Parallel (28 Gy, *k* = 4) |  | < 9.5 % |
| C | 6 | rectum | Parallel (18 Gy, *k* = 4) |  | < 34.5 % |
| C | 8 | femoral head Right | Maximum Dose |  | < 19 Gy |
| C | 9 | femoral head Left | Maximum Dose |  | < 19 Gy |
| P | 1 | urethral PRV (*a*) | Quadratic Overdose (35 Gy) |  | < 0.05 Gy |
| P | 2 | PTV_OVL_rectum (*b*) | Quadratic Overdose (34.5 Gy) | 0.0 cm (*a*) | < 0.10 Gy |
| P | 3 | PTV_OVL_bladder (*c*) | Quadratic Overdose (34.5 Gy) | 0.0 cm (*a,b*) | < 0.04 Gy |
| P | 4 | PTV (*d*) | Quadratic Overdose (37.3 Gy) | 0.0 cm (*a,b,c*) | < 0.02 Gy |
| P | 5 | External | Quadratic Overdose (32 Gy) | 0.0 cm (*a,b,c,d*) | < 0.09 Gy |
| P | 5 | External | Quadratic Overdose (11.5 Gy) | 5.1 cm (*a,b,c,d*) | < 0.02 Gy |

| Prioritized Objectives | | | | | |
| --- | --- | --- | --- | --- | --- |
| *Priority* | *Layering*  *Order* | *Structure* | *Cost Function*  *(parameter values)* | *Shrink margin (cm)* | *Goal* |
| 1 | 1 | urethral PRV (*a*) | Target Penalty (97%) |  | > 33.2 Gy |
| 1 | 2 | PTV_OVL_rectum (*b*) | Target Penalty (97%) | 1. cm (*a*) | > 33.2 Gy |
| 1 | 3 | PTV_OVL_bladder (*c*) | Target Penalty (95%) | 0.0 cm (*a,b*) | > 33.2 Gy |
| 1 | 4 | PTV (*d*) | Target Penalty (97%) | 0.0 cm (*a,b,c*) | > 35 Gy |
| 1 | 4 | PTV (*d*) | Target Penalty (99.5%) | 0.0 cm (*a,b,c*) | > 33.2 Gy |
| 2 | 6 | rectum | Parallel (28 Gy, *k* = 4) |  | < 1 % |
| 3 | 6 | rectum | Serial (*k* = 1) |  | < 10 Gy |
| 4 | 6 | rectum | Parallel (18 Gy, *k* = 4) |  | < 20 % |
| 5 | 7 | bladder | Serial (*k* = 1) |  | < 12 Gy |
| 6 | 5 | External | Conformality | 0.0 cm (*a,b,c,d*) | < 0.5 |
| 7 | 10 | penile bulb | Serial (*k* = 1) | 0.3 cm (*d*) | < 2 Gy |
| 8 | 8 | femoral head Right | Quadratic Overdose (15 Gy) |  | < 0.5 Gy |
| 8 | 9 | femoral head Left | Quadratic Overdose (15 Gy) |  | < 0.5 Gy |

*Layering order: order list according to which the shared voxels between distinct structures are assigned to distinct cost functions. Goal: goal values of the cost functions. Priority: order list according to which the objectives (cost functions) are optimized during mCycle 2^nd^ pass. Shrink margin: the minimum distance from the indicated structure for the voxels on which ones the cost function is applied.*
