# Supplementary material for: Automated Planning for Prostate Stereotactic Body Radiation Therapy on the 1.5 T MR-Linac
Source: Adv Radiat Oncol. 2022 Feb 12;7(3):100865. doi: 10.1016/j.adro.2021.100865 (PMC8850203; doi:10.1016/j.adro.2021.100865)
Supplement: Supplementary file 2 [file mmc2.docx]

	Structures	Dosimetric Criterion	Actual Values	
	Urethral PRV	V35Gy < 1 cm^3	0.009 cm^3	✓
		V33.2Gy > 95 %	100.00 %	✓
	PTV_OVL_rectum	V35Gy < 1 cm^3	0.541 cm^3	✓
		V33.2Gy > 95 % (-5 %)	98.37 %	✓
	PTV_OVL_bladder	V35Gy < 1 cm^3	0.467 cm^3	✓
		V33.2Gy > 95 % (-5 %)	99.73 %	✓
	PTV	V37.5Gy < 2 % (+1 %)	1.20 %	✓
		V33.2Gy > 95 % (-1 %)	99.11 %	✓
	rectum	V35Gy < 1 cm^3	0.315 cm^3	✓
		V32Gy < 5 % (+1 %)	3.83 %	✓
		V28Gy < 10 % (+1 %)	8.13 %	✓
		V18Gy < 35 % (+1 %)	20.35 %	✓
	bladder	V35Gy < 1 cm^3	0.459 cm^3	✓
	femural head_R	Dmax < 20 Gy (+1 Gy)	18.151 Gy	✓
	femural head_L	Dmax < 20 Gy (+1 Gy)	17.787 Gy	✓
	Penile bulb	V35Gy < 1 cm^3	0.000 cm^3	✓
	PTV_r&b&uPRV	V37.5Gy < 2 % (+1 %)	1.31 %	✓
		V33.2Gy > 95 % (-1 %)	98.73 %	✓

FIGURE 1S: an example of the translation of the necessary dosimetric criteria for planning approval into scorecards, as performed by Monaco^{TM} TPS.
