# Supplementary material for: Automated Planning for Prostate Stereotactic Body Radiation Therapy on the 1.5 T MR-Linac
Source: Adv Radiat Oncol. 2022 Feb 12;7(3):100865. doi: 10.1016/j.adro.2021.100865 (PMC8850203; doi:10.1016/j.adro.2021.100865)
Supplement: Supplementary file 3 [file mmc3.docx]

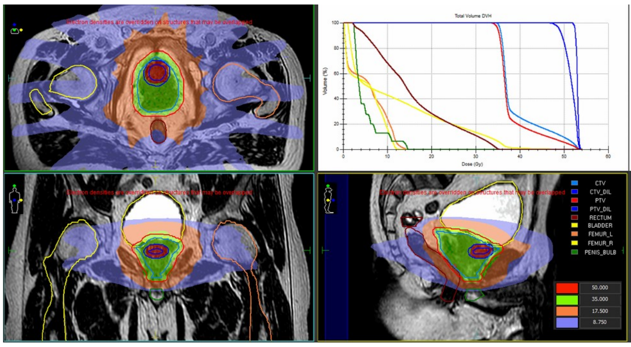

	Structure	Dosimetric Criterion	Actual Value
	CTV_DIL	V50Gy > 99 %	100.00 %
	PTV_DIL	V53.5Gy < 2 %	1.19 %
		V47.5Gy > 99 % (-1 %)	99.44 %
	PTV	V33.2Gy > 99 % (-1 %)	99.98 %
	RECTUM	V38Gy < 1 cm³	0.000 cm³
		Dmax < 40 Gy	35.611 Gy
		V35Gy < 2 cm³	0.159 cm³
		V32Gy < 15 %	7.48 %
		V28Gy < 20 %	14.17 %
	BLADDER	V37Gy < 10 cm³	5.405 cm³
		V32Gy < 15 %	11.05 %
		V28Gy < 20 %	15.83 %
	PENIS_BULB	V20Gy < 90 %	0.00 %
	FEMURS	V28Gy < 5 %	0.00 %

Figure 2S: an mCycle plan, at 35Gy/5fx to the prostate with a SIB to 50Gy to the dominant intraprostatic lesion (DIL). Dose distributions on the three normal planes, DVH curves, and the resulting values for the metrics adopted as dose criteria for planning approval, according to HypoFLAME trial (NCT02853110), are reported.
